# Adaptive demodulation by deep-learning-based identification of fractional orbital angular momentum modes with structural distortion due to atmospheric turbulence

**DOI:** 10.1038/s41598-021-03026-z

**Published:** 2021-12-06

**Authors:** Youngbin Na, Do-Kyeong Ko

**Affiliations:** Department of Physics and Photon Science, Gwangju Institute of Science and Technology, Gwangju, 61005 Republic of Korea

**Keywords:** Optical physics, Fibre optics and optical communications

## Abstract

Since the great success of optical communications utilizing orbital angular momentum (OAM), increasing the number of addressable spatial modes in the given physical resources has always been an important yet challenging problem. The recent improvement in measurement resolution through deep-learning techniques has demonstrated the possibility of high-capacity free-space optical communications based on fractional OAM modes. However, due to a tiny gap between adjacent modes, such systems are highly susceptible to external perturbations such as atmospheric turbulence (AT). Here, we propose an AT adaptive neural network (ATANN) and study high-resolution recognition of fractional OAM modes in the presence of turbulence. We perform simulations of fractional OAM beams propagating through a 1-km optical turbulence channel and analyze the effects of turbulence strength, OAM mode interval, and signal noise on the recognition performance of the ATANN. The recognition of multiplexed fractional modes is also investigated to demonstrate the feasibility of high-dimensional data transmission in the proposed deep-learning-based system. Our results show that the proposed model can predict transmitted modes with high accuracy and high resolution despite the collapse of structured fields due to AT and provide stable performance over a wide SNR range.

## Introduction

The application of an orbital angular momentum (OAM) as an additional degree of freedom for information transfer has been a popular topic in optical communications in recent years^1–8^. Light OAM associated with the spatial distribution stems from a helical phase $\mathrm{exp}\left(il\varphi \right)$, where $l$ is a topological charge (TC), and $\varphi$ is the azimuth angle in the cylindrical coordinate system^9,10^. The so-called “twisted” light with these structured phases carries an OAM of $l\hslash$ per photon^9^, and the TC, which determines the OAM mode, takes an integer or fractional value^11–13^. Unlike spin angular momentum with only two possible values, theoretically unbounded values of the OAM can provide high degrees of freedom^1^. Indeed, many experimental results have shown that the transmission capacity can be improved beyond Tbit/s with integer OAM beams along with polarization- and wavelength-division multiplexing^1,2,4,5^. However, there are challenges to be overcome, such as diffraction effects of higher-order modes^14^ (beam size and beam divergence) and the space-bandwidth product of a given optical system^6,15^. The number of addressable OAM modes is physically restricted by the system, even though the range of the OAM mode itself is unbounded^6,15^. Besides, the higher-order mode with a larger beam size is relatively more vulnerable to atmospheric distortion, resulting in higher modal crosstalk^16^. Hence, OAM modes separated by fractional intervals have drawn attention as a potential solution to the problems. One could exploit more OAM modes under fewer physical resources (space-bandwidth product of the system) by reducing the mode interval as small as possible. Indeed, recent studies have shown the tremendous potential of fractional OAM links combined with superhigh-resolution recognition techniques based on a deep-learning model^17–19^.

Artificial neural networks comprised of multiple processing layers have been widely applied as a precise, efficient tool for detecting OAM modes^17–24^. Deep-learning models directly recognize transmitted spatial modes without any optical mode sorter for extracting phase information^18,20^ and provide reliable performance against distorting factors such as optical misalignment^18,24,25^. Adaptive demodulation^21–23^ and turbulence correction^26,27^ for integer vortex beams have been studied in depth by pioneering researchers. Meanwhile, the previous studies on fractional OAM modes focused on high-resolution recognition and the capability to increase communication capacity^17,18^. The researchers have experimentally demonstrated that a deep-learning model based on a convolutional neural network can classify fractional OAM modes with a resolution of up to 0.01^17^ and process two independent spatial degrees of freedom simultaneously^18^. Jing et al*.* built a deep neural network for recognizing fractional OAM modes^19^. The author improved anti-turbulence ability by combining a method of diffraction preprocessing of a two-dimensional fork grating and achieved the recognition accuracy of 99.1% for the mode interval of 0.1. In addition, deep-learning-based detection of hybrid beams carrying fractional topological charge and the fractional angular ratio was investigated, which showed accurate recognition of fractional OAM with broad bandwidth in atmospheric environments^28^.

Despite the remarkable success of the deep-learning scheme, there is the problem of long-distance transmission and detection of fractional OAM beams. Atmospheric turbulence (AT) and power loss, i.e., low SNR, are the most problematic factors. The AT, which results from random variations in air refractive index, leads to adversarial effects on the propagating structured light, distorting a light wavefront and degrading the mode purity^29,30^. In particular, at long-distance propagation where phase distortion accumulates, such adversarial effects become more apparent. Compared to the integer OAM mode of the ring structure, the peculiar spatial distribution of the fractional OAM mode can provide various local features for effective classification^11–13,18^. However, optical systems employing fractional OAM modes are expected to be highly sensitive to external perturbations due to tiny structural differences between adjacent modes. Moreover, the recognition performance would be much affected by the selected mode interval. Therefore, for the practical application of such OAM modes, it is necessary to develop an optical system capable of performing high-resolution recognition regardless of distorting factors.

Here, we build an AT adaptive neural network called ATANN for high-resolution recognition of fractional OAM modes transmitted through optical turbulence channels. To prepare data sets for training and testing, we first model 1-km optical turbulence channels consisting of random phase screens that mimic turbulence environments. Then, we simulate the beam propagation and analyze the effects of turbulence level, signal noise, and mode intervals between fractional OAM modes on the model performance. In particular, the recognition accuracy of the ATANN is investigated for 5 kinds of 10-ary OAM shift keying systems, respectively, with mode intervals of 0.05, 0.10, 0.15, 0.20, and 0.25. We also discuss the generalization performance of the deep-learning method and the recognition of multiplexed fractional OAM beams.

## Method

### Fractional OAM beams evolving from spiral phase plates

Light beams carrying an OAM originate from a helical phase structure^11^

1 $$\begin{array}{c}{\psi }_{l}\left(r,\varphi \right)=exp\left(il\varphi \right),\end{array}$$

where $l$ is the azimuthal mode index that determines the TC of output fields, and $\left(r,\varphi \right)$ are the transverse coordinates in the cylindrical coordinate system. For integer $l$ values, the phase front of such beams forms $l$ intertwined helical structure and the consequent phase singularity, creating doughnut shape intensity distribution^11^. Meanwhile, for fractional $l$ values, the phase is no longer an integer multiple of $2\pi$ and creates a phase discontinuity along the radial direction, resulting in a symmetry-broken complex-phase structure^11,31^. The propagation of fractional OAM beams evolving from a spiral phase is calculated numerically by the angular spectrum method, which can be written as^32^

2 $$\begin{array}{c}{U}_{l}\left(\rho ,\theta ,\Delta z\right)={FFT}^{-1}\left\{FFT\left\{{u}_{l}\left(r,\varphi ,0\right)\right\}\mathrm{exp}\left[i\Delta z\sqrt{{k}^{2}-4{\pi }^{2}\left({f}_{x}^{2}+{f}_{y}^{2}\right)}\right]\right\}, \end{array}$$

where $\left(\rho ,\theta \right)$ are the cylindrical coordinate components of the output plane, $\Delta z$ is the propagation distance, $FFT \left({FFT}^{-1}\right)$ represents the 2D fast Fourier transform operation, $k$ is the wavenumber, and $\left({f}_{x},{f}_{y}\right)$ are the spatial frequency components. The second term in the inverse fast Fourier transform operation corresponds to the angular spectrum transfer function. ${u}_{l}\left(r,\varphi ,0\right)={u}_{\mathrm{g}}\left(r,\varphi ,0\right){\psi }_{l}\left(r,\varphi \right)$ is the field distribution in the input plane, where ${u}_{\mathrm{g}}$ represents an incident Gaussian beam. Figure 2 (top row) shows 10 fractional OAM beams separated by a fractional interval of 0.10 as an example. As shown in Fig. 2, fractional TCs give rise to local variations in the spatial field distribution, allowing a deep-learning model to discriminate them effectively despite the small mode interval^18^. Here, we assume 5 types of 10-OAM free-space optical links, where each of them has different mode spacing $\Delta l$ among $\left\{0.05, 0.10, 0.15, 0.20, 0.25\right\}$. 10 OAM modes composing each link are presented in Table 1.

**Table 1:** 5 mode intervals and the corresponding 10 OAM modes.

Spacing	OAMs
0.05	1.10–1.55
0.10	1.10–2.00
0.15	1.10–2.45
0.20	1.10–2.90
0.25	1.10–3.35

### Simulation of atmospheric turbulence channels

**Figure 1:**
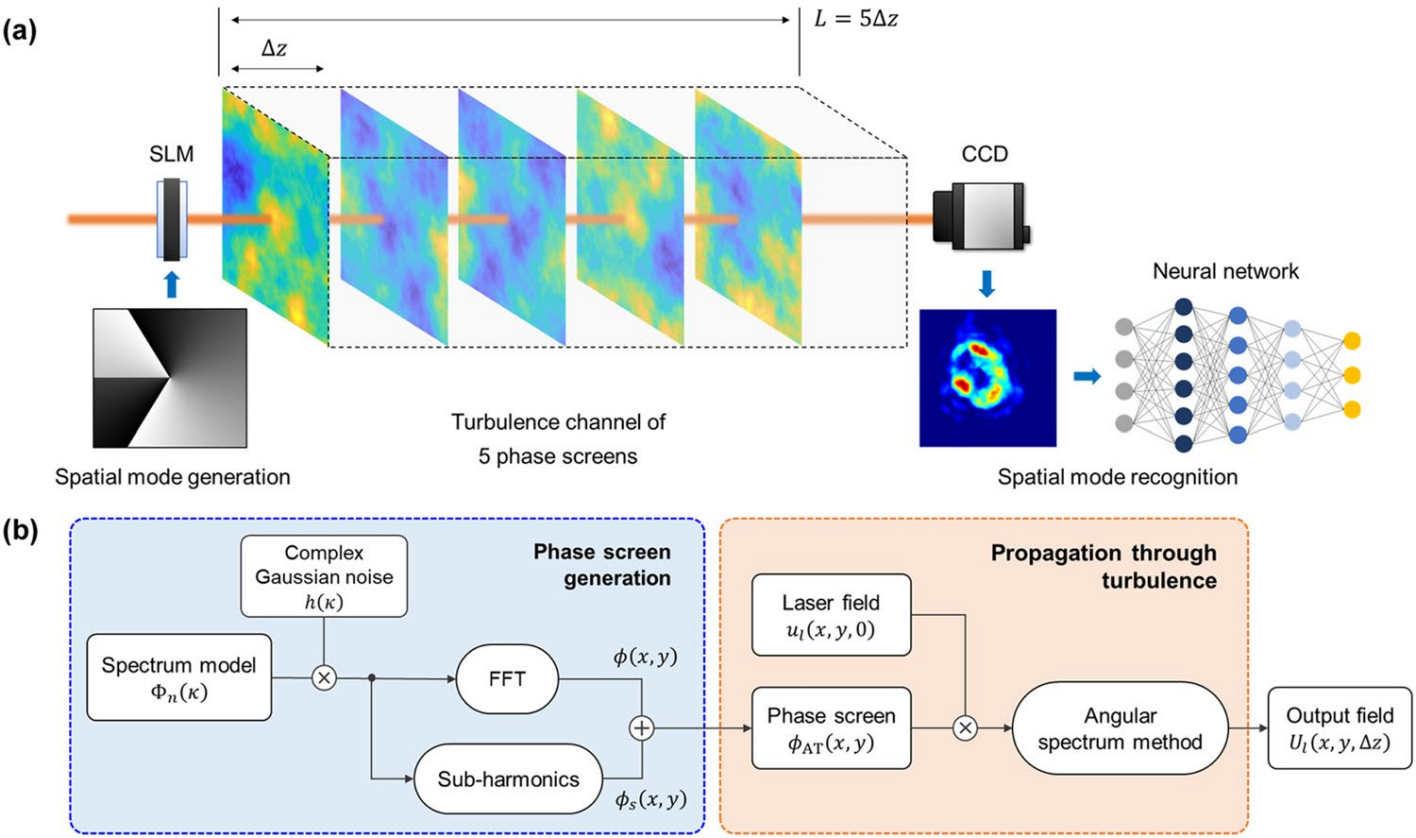
Schematic diagrams for investigating AT effects. (**a**) Geometry of the simulation model and (**b**) a flowchart for numerical simulation of laser beams propagating through AT media. Each inset in (**a**) displays the phase hologram (left) and intensity profile (right) of fractional OAM mode $l=1.50$, respectively.

Simulation of laser beams propagating through AT channels is implemented using the split-step Fourier method and random phase plates placed along the propagation direction^16,32–34^. As shown in Fig. 1a, the turbulent channel with a total link distance of 1 km is modeled with 5 phase screens separated by distance $\Delta z=200\,\mathrm{m}$. Here, each screen imposes the integrated phase of turbulence that exists over the distance $\Delta z$. The propagation of laser beams is calculated with the procedure shown in Fig. 1b. First, an OAM beam ${u}_{l}\left(x,y,0\right)$ incident upon the first screen is multiplied by the phase function $\mathrm{exp}\left[i{\phi }_{\mathrm{AT}}\left(x,y\right)\right]$. Then, it propagates to the next screen, which is implemented using the angular spectrum method described in Eq. (2). The difference here is that the input field distribution ${u}_{l}\left(x,y,0\right)$ turns into ${u}_{l}\left(x,y,0\right)\mathrm{exp}\left[i{\phi }_{\mathrm{AT}}\right]$. The output field ${U}_{l}\left(x,y,\Delta z\right)$ is used again as the field incident upon the second one, and the process is repeated until the total distance $L$ is reached. After that, the final field distribution is measured at the receiver.

The random phase screens that mimic the AT-induced distortion effect are produced by the power spectrum of refractive index fluctuations with statistical properties similar to those of a turbulent environment^30^. A variety of spectrum models have been presented to emulate the AT^29^. The most representative model is the Kolmogorov power-law spectrum expressed as^29^

3 $$ {\Phi }_{n} \left( {\kappa_{x} ,\kappa_{y} } \right) = 0.033C_{n}^{2} \left( {\kappa_{x}^{2} + \kappa_{y}^{2} } \right)^{ - 11/6} , $$

where $\left({\kappa }_{x},{\kappa }_{y}\right)$ is the angular spatial frequency, and ${C}_{n}^{2}$ is the structure constant of the refractive index that determines the turbulence strength. It is widely used in theoretical and numerical studies due to its relatively simple mathematical form. However, the spectrum only applies to the inertial subrange of $1/{L}_{0}\ll \kappa \ll 1/{l}_{0}$, where ${l}_{0}$ and ${L}_{0}$ are the inner and outer scales of turbulence, and is not able to describe some physical phenomena such as the small bump at high wavenumber near $1/{l}_{0}$ ^29^. Therefore, we use the modified atmospheric spectrum, developed by Hill^35^ and defined analytically by Andrew^36^. It can provide tractability for theoretical studies and explain the phenomenon occurring at the high wavenumber. The spectrum is expressed as^29^

4 $$\begin{aligned} &{\Phi }_{n}\left({\kappa }_{x},{\kappa }_{y}\right)=0.033{C}_{n}^{2}\left[1+1.802{\left(\frac{{\kappa }_{x}^{2}+{\kappa }_{y}^{2}}{{\kappa }_{l}^{2}}\right)}^\frac{1}{2}-0.254{\left(\frac{{\kappa }_{x}^{2}+{\kappa }_{y}^{2}}{{\kappa }_{l}^{2}}\right)}^\frac{7}{12}\right]\\ &\times \frac{\mathrm{exp}\left[-\left({\kappa }_{x}^{2}+{\kappa }_{y}^{2}\right)/{\kappa }_{l}^{2}\right]}{{\left({\kappa }_{x}^{2}+{\kappa }_{y}^{2}+{\kappa }_{0}^{2}\right)}^{11/6}},\end{aligned}$$

where ${\kappa }_{l}=3.3/{l}_{0}$, and ${\kappa }_{0}=2\pi /{L}_{0}$. Here, we assume that the structure constant ${C}_{n}^{2}$ is constant throughout the optical channel. Meanwhile, the variance of the phase spectrum is given by^32,33^

5 $$\begin{array}{c}{\sigma }^{2}\left({\kappa }_{x},{\kappa }_{y}\right)={\left(\frac{2\pi }{N\Delta x}\right)}^{2}2\pi {k}^{2}\Delta z{\Phi }_{n}\left({\kappa }_{x},{\kappa }_{y}\right),\end{array}$$

where $N$ and $\Delta x$ represent the number of grid points and the spatial grid interval, respectively. The random realization of the AT phase screen is implemented by multiplying a complex Gaussian random matrix and then performing the FFT operation, which can be written as^32,33^

6 $$\begin{array}{c}\phi \left(x,y\right)=FFT\left\{{h}_{N\times N}\sigma \left({\kappa }_{x},{\kappa }_{y}\right)\right\},\end{array}$$

where ${h}_{N\times N}$ is a complex Gaussian random matrix with mean 0 and variance 1. Additionally, a subharmonic method is used to compensate for under-sampling for low-frequency components that induce beam wander^32,33^. The method is to resample the spectrum near the origin with sub-grid points and incorporate a phase derived from that region into the phase screen $\phi \left(x,y\right)$. Thus, the final phase screen used for the simulation is given as ${\phi }_{\mathrm{AT}}=\phi +{\phi }_{s}$, where ${\phi }_{s}$ is the subharmonic screen.

To investigate the effects of AT strength, we prepared data sets for 5 different turbulence levels ${C}_{n}^{2}\left[{\mathrm{m}}^{-2/3}\right]=\left\{1\times {10}^{-16}, 5\times {10}^{-16},1\times {10}^{-15},5\times {10}^{-15},1\times {10}^{-14}\right\}$. Here, we selected the range of ${C}_{n}^{2}$ and the sampling distance $\Delta z$ by considering the conditions for the Rytov variance^16,32^, ${\sigma }_{R}^{2}\left(L\right)<1$ and ${\sigma }_{R}^{2}\left(\Delta z\right)<0.1{\sigma }_{R}^{2}\left(L\right)$. The random phase screen is set with window size ${L}_{x}={L}_{y}=0.8\,\mathrm{m}$, the number of grid points $N=1024$, grid interval $\Delta x=\Delta y={L}_{x}/N$, inner scale ${l}_{0}=2\,\mathrm{mm}$, and outer scale ${L}_{0}=50\,\mathrm{m}$. A laser beam with wavelength $\uplambda =594\,\mathrm{nm}$ and beam radius ${w}_{0}=0.03\,\mathrm{m}$ is used as the light source. The central 200 × 200 area of the simulation window is set as the observation region. All intensity profiles measured in that region are preprocessed for supervised learning and resized from 200 × 200 to 80 × 80 pixels for computational efficiency.

**Figure 2:**
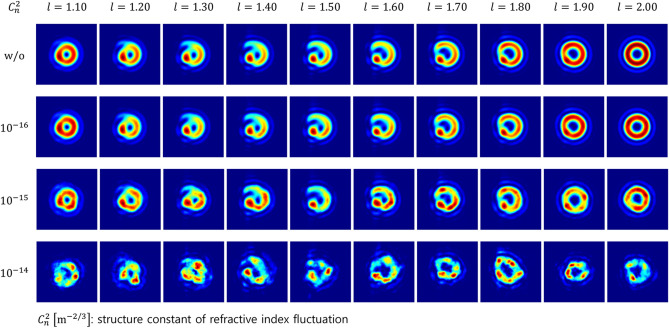
Intensity profiles of the received fractional OAM beams over 1000-m AT channels with different ${C}_{n}^{2}$. Here, each image is 200 × 200 pixels wide.

Figure 2 shows intensity profiles of 10 fractional OAM beams transmitted through 1000-m AT channels with different values of ${C}_{n}^{2}$. For the turbulence with ${C}_{n}^{2}\le 1\times {10}^{-15} \,{\mathrm{m}}^{-2/3}$, each OAM beam well preserves the local structure that differentiates itself from others, so it is expected that the deep-learning model recognizes transmitted OAM modes with high accuracy despite the small spacing $\Delta l=0.05$. For the strong turbulence with ${C}_{n}^{2}=1\times {10}^{-14}\, {\mathrm{m}}^{-2/3}$, the local features are severely destroyed. However, the surprising thing is that the ATANN can, even in this case, extract the inherent features of each fractional mode and perform classification; see Fig. 7c. Here, we used the pseudo color for visual clarity of beam profile images, but the actual image format input to the neural network is 8-bit grayscale.

### Architecture of the ATANN

**Figure 3:**
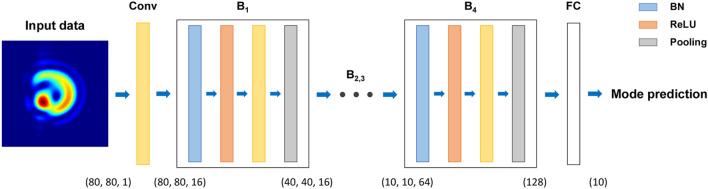
Schematic diagram of the ATANN: Conv, convolutional layer; B, convolution block; BN, batch normalization layer; FC, fully connected layer. Each block consists of BN, ReLU, Conv, and Pooling layers.

Deep learning is a subset of artificial intelligence inspired by the structure and the mechanism of the human brain. It uses an artificial neural network with multiple processing layers in order to learn representations of data with high levels of abstraction^37^. The architecture of ATANN is depicted schematically in Fig. 3; see Table 2 for details. It comprises a series of convolution blocks for feature extraction and one fully connected (FC) layer for mode classification. Each block consists of four types of layers: batch normalization (BN)^38^, rectified linear unit (ReLU)^39^, convolution, and pooling. The BN is a layer that normalizes each feature map for the input mini-batch, maintaining the mean and standard deviation of the output close to 0 and 1. The purpose of BN is to accelerate the training of neural networks while mitigating overfitting^38^. To utilize BN effectively, we adopted the so-called pre-activation structure of BN-ReLU-Conv presented in modern network architectures such as residual network (ResNet)^40^ and densely connected network (DenseNet)^41^. The ReLU is a nonlinear activation function of the form $f\left(z\right)=\mathrm{max}\left(z,0\right)$, whose purpose is to provide non-linearity to the output^18^. The ReLU is suitable for deep neural networks possessing many hidden layers because it can solve the gradient vanishing problem^39^. Multiple convolution filters in a convolutional layer are convolved with the input image and generate various feature maps. The extracted feature maps are downsampled in half by a max-pooling layer with a 2 × 2 window^42^, and the downsampled feature maps are fed into the next block. Note that the last block uses a global max-pooling layer that extracts only one maximum value for each feature map; see Table 2. Compared to the max-pooling layer followed by the flatten operation^18^, it significantly reduces the number of trainable parameters, which is computationally efficient and more effective for alleviating overfitting. After the extraction process is complete, the FC layer predicts a received mode by integrating the output of the last block and applying a softmax activation function.

**Table 2:** Detailed information on the ATANN.

	Layer	Hyperparameter	Output shape	# of parameters
Conv	Conv2D	16, (3, 3), padding	(80, 80, 16)	160
B_1_	BatchNorm	0.3	(80, 80, 16)	64
	Activation	ReLU	(80, 80, 16)	0
	Conv2D	16, (3, 3), padding	(80, 80, 16)	2,304
	Maxpool2D	(2, 2)	(40, 40, 16)	0
B_2_	BatchNorm	0.3	(40, 40, 16)	64
	Activation	ReLU	(40, 40, 16)	0
	Conv2D	32, (3, 3), padding	(40, 40, 32)	4,608
	Maxpool2D	(2, 2)	(20, 20, 32)	0
B_3_	BatchNorm	0.3	(20, 20, 32)	128
	Activation	ReLU	(20, 20, 32)	0
	Conv2D	64, (3, 3), padding	(20, 20, 64)	18,432
	Maxpool2D	(2, 2)	(10, 10, 64)	0
B_4_	BatchNorm	0.3	(10, 10, 64)	256
	Activation	ReLU	(10, 10, 64)	0
	Conv2D	128, (3, 3), padding	(10, 10, 128)	73,728
	GlobalMaxpool2D	–	(128)	0
–	Dropout	20%	(128)	0
FC	Dense	10, softmax	(10)	1,290

## Results and discussion

### Training of neural network and recognition performance

Training a neural network is to find the optimized weights of trainable layers, i.e., parameters of convolutional layers and FC layers, with a gradient descent algorithm. In this study, we prepared a total of 25 data sets, taking into account 5 turbulence levels and 5 OAM intervals, as described above. Each data set contains 700 (500 for training, 100 for validation, and 100 for testing) intensity profile images per mode, i.e., 7,000 intensity profiles in total. Model training and testing are implemented on a commercial laptop system (CPU: i7-9750H; GPU: RTX 2060) based on the Keras framework. We run the training with batch size 20 for 50 epochs, and the weights of the ATANN are updated automatically with the Adam optimizer^43^, a stochastic optimization method. The optimization process is implemented by minimizing the loss function, which is expressed as^17^

7 $$\begin{array}{c}L=-\frac{1}{N}\sum_{i=1}^{N}\sum_{j=1}^{m}{y}_{j}^{(i)}\mathrm{log}\left(\frac{{z}_{j}^{(i)}}{{\sum }_{n=1}^{m}exp\left({z}_{n}^{(i)}\right)}\right),\end{array}$$

where $N$ and $m$ represent the number of samples and the number of OAM modes, respectively, and $j$ represents the index corresponding to each OAM mode. ${y}_{j}^{(i)}$ is the *j*th element of a 1D label vector of size $m$. An element of the vector is 1 if the index $j$ corresponds to a target OAM mode and 0 otherwise. For example, for the 10-OAM with $\Delta l=0.10$, label vectors of the OAM mode of $l=1.10$ and $l=2.00$ are $\left\{1, 0, \dots , 0\right\}$ and $\left\{0, 0, \dots , 1\right\}$, respectively. Note that the function in the parentheses corresponds to the operation of the softmax function, which yields the probabilities that the input samples belong to each OAM mode^18^. Here, ${z}_{j}$ is the output of the *j*th neuron in the FC layer, which is calculated as a weighted sum of its inputs from the previous layer^37^. Meanwhile, the learning rate, initially set to be 0.001, is set to decrease by a factor of 0.5 if no improvement in the validation loss is seen for 5 epochs.

**Figure 4:**
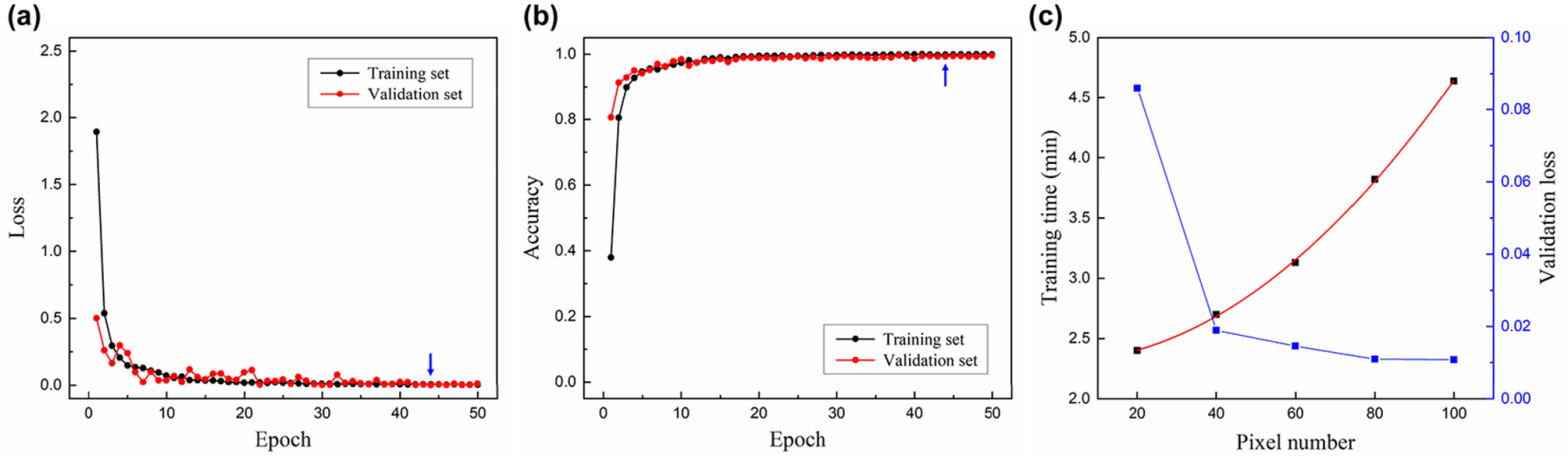
Training of the ATANN. (**a**) Loss and (**b**) accuracy curves for a training set and a validation set (${C}_{n}^{2}=1\times {10}^{-14}\, {\mathrm{m}}^{-2/3}$ and $\Delta l=0.20$). (**c**) Model evaluation for the input pixel size. Blue arrows indicate 44th epoch at which minimum validation loss was achieved. Each data displayed in (**c**) is the result of a data set (${C}_{n}^{2}=1\times {10}^{-14} \,{\mathrm{m}}^{-2/3}$ and $\Delta l=0.15$) and shows the mean value of 15 training results. The red curve in (c) represents a fitting line.

Figure 4a,b show the training results of the proposed ATANN, as an example, for the data set (${C}_{n}^{2}=1\times {10}^{-14} \,{\mathrm{m}}^{-2/3}$ and $\Delta l=0.20$). Here, the loss is the value calculated by Eq. (7), and the accuracy is defined as the number of correctly recognized samples out of the total number of samples. As the training progresses, the ATANN discovers proper weights for classifying fractional modes, and accordingly, the loss function decreases and gradually converges. Meanwhile, since only the training set participates in the model training, there may be weak fluctuations in the loss of the validation set compared to the training set, as shown in Fig. 4a. Nevertheless, the validation curve follows the training curve well without overfitting, which indicates that the trained model can perform predictions even on new data. After the training is complete, optimized weights that minimize the loss of the validation set are stored and then used to test the recognition performance.

Once the architecture and training of a neural network are complete, the computational speed is determined by the size of input data^18^ and the Floating-point Operations Per Second (FLOPS) of the computing resources^27^, i.e., GPU or CPU. The FLOPS is a value fixed by the given computing system, so only the effect of the number of pixels is investigated. Figure 4c shows the training time and validation loss for the input pixel size. Intensity profiles, resized from 200 × 200 to 5 different pixel numbers, are used to train the ATANN. The time of the model training increases in proportion to the square of the number of pixels. On the other hand, the measured loss function is high at 20 × 20 pixels but decreases with increasing the resolution and converges to about 0.01 after 60 × 60 pixels. The result of 80 × 80 pixels shows a performance relatively lower than the higher resolution (100 × 100). However, for application fields such as optical communications, both the demodulation performance and the computational speed are critical factors, so a resolution of 80 × 80 pixels is suitable for our task. For input images of 80 × 80 pixels, the measured prediction time is less than 3 ms, which can be reduced further by a high-performance GPU with faster FLOPS^27^.

**Figure 5:**
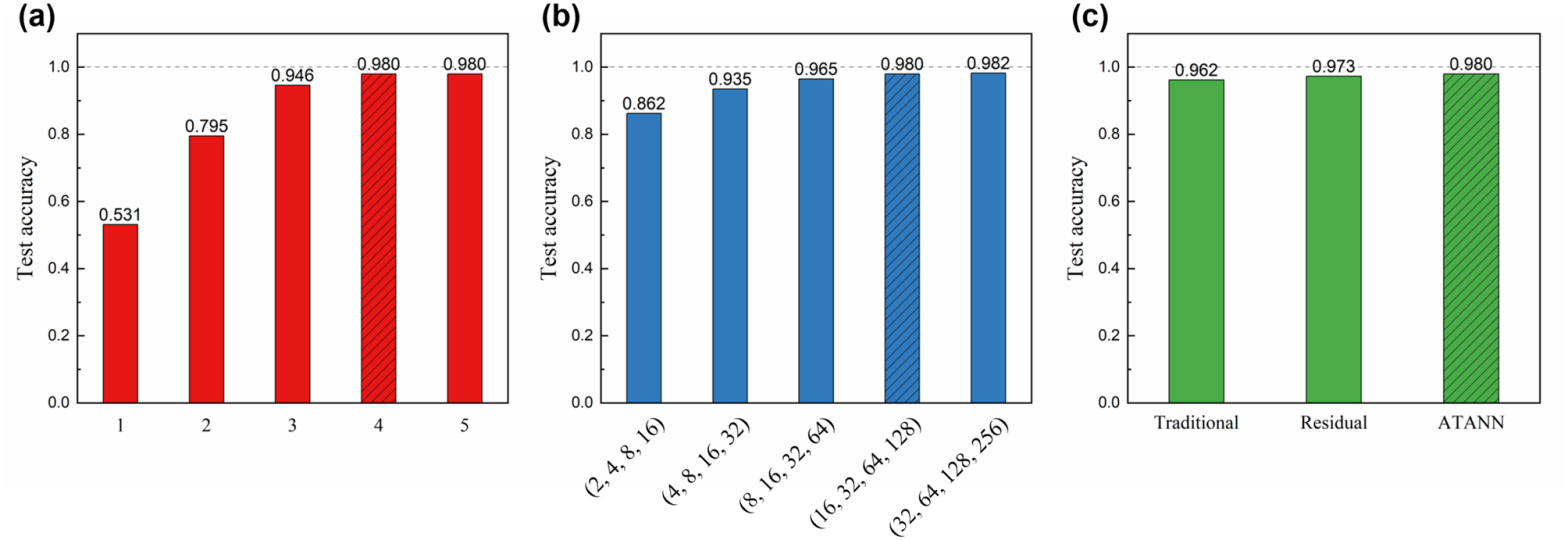
Comparison of the recognition performance for model architectures. Influence of (**a**) the number of convolution blocks and (**b**) the number of filters in convolutional layers. Recognition performance for models with different configurations of convolution blocks. All data presented here are the result for a data set (${C}_{n}^{2}=1\times {10}^{-14}\, {\mathrm{m}}^{-2/3}$ and $\Delta l=0.15$) and represent the mean values of 5 test results. Hatched bars in each bar graph represent results of the proposed ATANN.

Before we continue, we compared the proposed ATANN with different architectures of networks to check whether the designed system is appropriate. Here, we investigated the influence of three main hyperparameters that determine the model architecture: (1) the number of used convolution blocks, (2) the number of filters in convolutional layers, and (3) the configuration of convolution blocks constituting a neural network. First, we investigated the influence of the number of convolution blocks on the recognition accuracy. As shown in Fig. 5a, the accuracy of the neural network improves from 53.1 to 98.0% as the number of convolution blocks increases from 1 to 5. Models composed of 4 or 5 convolution blocks showed the optimized performance of 98.0%, and we selected four-block architecture. The influence of the number of filters in convolutional layers is illustrated in Fig. 5b. Here, filters of convolutional layers placed in the convolution blocks were adjusted. Similar to the case of the number for blocks, increase of the number of filters improves model performance, which is because the trained model can learn more intrinsic features from the input data^42^. Although the model with (32, 64, 128, 256) filters shows relatively higher accuracy, it has about 3.9 times more trainable parameters, which slows down the computation. Therefore, the number of filters of the ATANN was set as (16, 32, 64, 128) for efficiency.

Figure 5c displays the recognition performance of neural networks with different configuration of convolution blocks. “Traditional” corresponds to the well-known AlexNet configuration of Conv-ReLU-Pooling, and “Residual” stands for residual unit^40^, a structure with skip connection. Meanwhile, our proposed ATANN consists of pre-activation structure BN-ReLU-Conv-Pooling. Here, to minimize the difference in the number of weight parameters due to configurations, the number of blocks constituting a neural network and the number of convolution filters were set to be the same. Compared with the traditional framework, we can see that adding the BN increases recognition performance from 96.2% to 98.0%, indicating better generalizability without overfitting. The result shows that the ATANN comprised of convolution blocks of pre-activation configuration is more effective for our problem.

**Figure 6:**
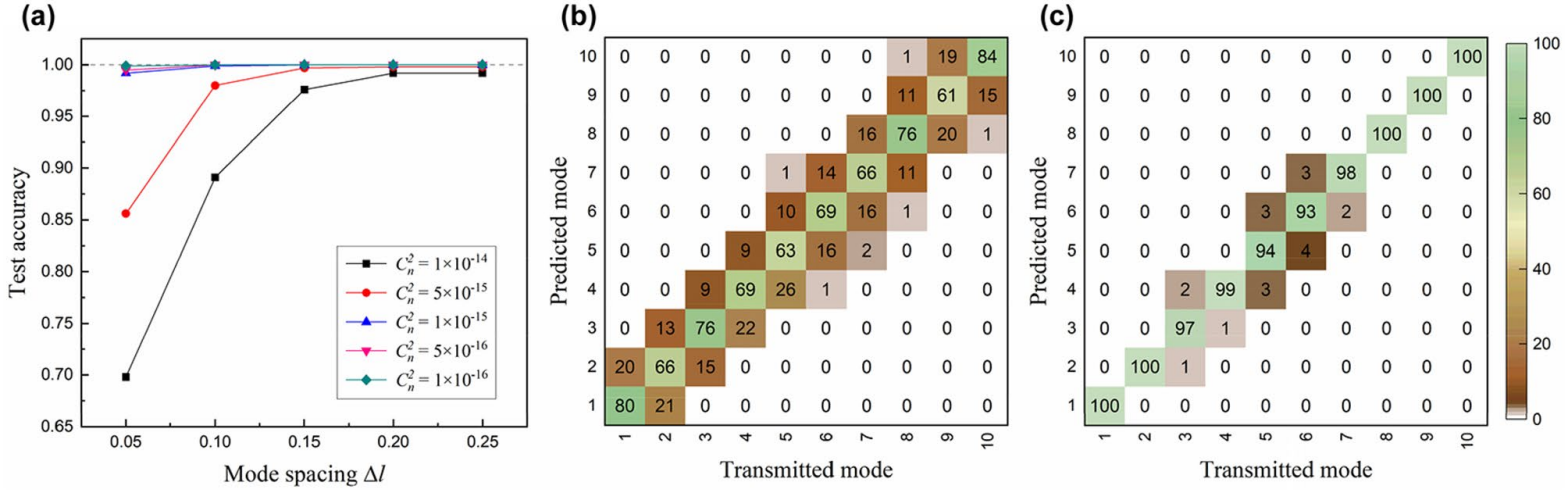
Influence of OAM mode interval $\Delta l$ and turbulence strength ${C}_{n}^{2}$ on recognition performance. (**a**) Recognition accuracy as a function of OAM mode spacing and (**b**,**c**) crosstalk matrices for a 1000-m link with ${C}_{n}^{2}=1\times {10}^{-14}\, {\mathrm{m}}^{-2/3}$. Here, (**b**) and (c) correspond to the results of $\Delta l=0.05$ (10 OAM modes from 1.10 to 1.55) and $\Delta l=0.15$ (10 OAM modes from 1.10 to 2.45), respectively. All data presented in (**a**) correspond to the mean values of 5 test results per data set.

To investigate the recognition performance of the trained ATANN, we conduct mode prediction with test data sets and analyze the recognition accuracy. Figure 6 shows the influence of turbulence level and mode spacing on the recognition accuracy. For the turbulence with ${C}_{n}^{2}\le 1\times {10}^{-15} \,{\mathrm{m}}^{-2/3}$, the recognition accuracy was measured to be over 99.2% regardless of mode spacing, which means that the ATANN is able to identify transmitted fractional OAM modes up to a resolution of 0.05 under that turbulence condition. For the strong turbulence with ${C}_{n}^{2}\ge 5\times {10}^{-15}\,{\mathrm{m}}^{-2/3}$, the recognition performance for $\Delta l=0.05$ is severely degraded, and the accuracy drops to below 70%. It is because distortion that exceeds the decision boundaries of the ATANN causes a lot of wrong predictions; see Fig. 6b. Mode prediction of the ATANN is performed by $\mathrm{argmax}\left(\bullet \right)$, an operation that selects an element (index *i*) possessing the maximum probability ${z}_{i}/{\sum }_{n}\mathrm{exp}\left({z}_{n}\right)$. To achieve this, our model finds the optimized weight parameters through the training process, and the parameters act as the decision boundary for classifying 10 fractional OAM modes. Meanwhile, as the mode spacing increases, the recognition accuracy improves rapidly, showing reduced crosstalk between adjacent modes; see Fig. 6c. The recognition accuracy reaches 99.2% at ${C}_{n}^{2}=1\times {10}^{-14}\, {\mathrm{m}}^{-2/3}$ and $\Delta l=0.20$. In other words, it is possible to build a stable measurement system by selecting the appropriate mode interval for a given turbulence environment. The application of AT compensation techniques could lower the resolution limited by turbulence further.

**Figure 7:**
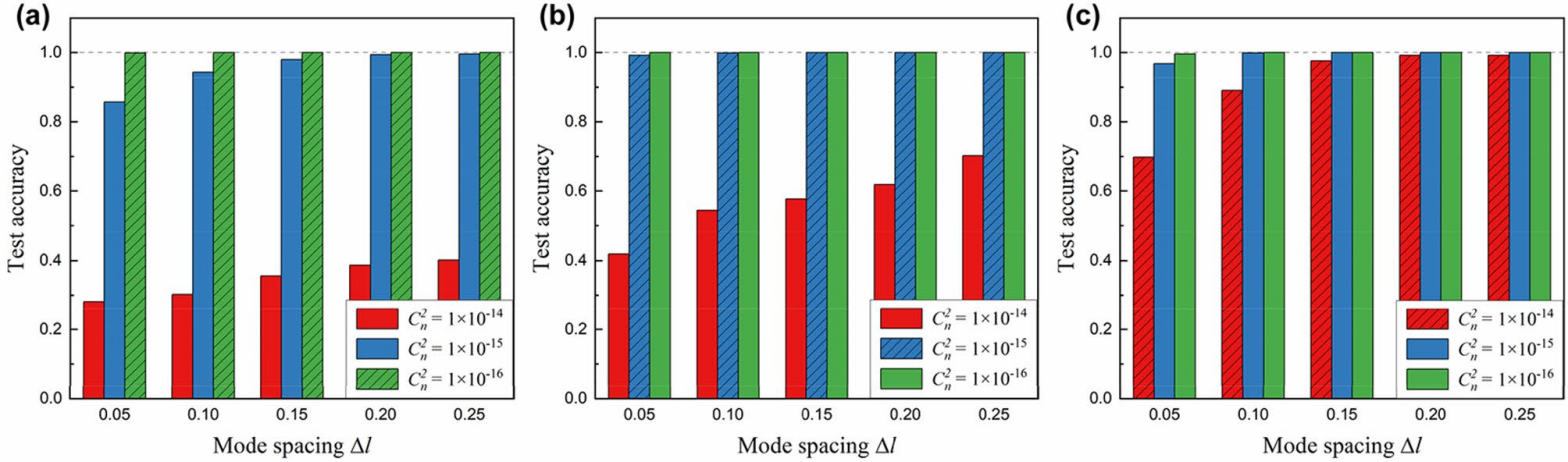
Generalization ability under unknown turbulence environments. Recognition performance for models trained with a data set of (**a**) ${C}_{n}^{2}=1\times {10}^{-16} \,{\mathrm{m}}^{-2/3}$, (**b**) ${C}_{n}^{2}=1\times {10}^{-15} \,{\mathrm{m}}^{-2/3}$, and (**c**) ${C}_{n}^{2}=1\times {10}^{-14} \,{\mathrm{m}}^{-2/3}$. Hatched bars in each bar graph represent prediction results obtained from test sets with the same AT strength ${C}_{n}^{2}$ as the training sets. Each data shows the mean value of 5 test results.

Until now, we have tested the model performance at the same turbulence strength as the training data, i.e., the pre-learned turbulence level. However, actual turbulence is not a static phenomenon, and its strength is not constant. Thus, it is necessary to investigate the generalization ability of the ATANN for unknown turbulence levels. Here, the “unknown” stands for a case that the turbulence strength ${C}_{n}^{2}$ considered in the test set is different from that of the training set. Figure 7a–c show the generalization performance of models trained with 3 different data sets, whose ${C}_{n}^{2}$ is $1\times {10}^{-16} \,{\mathrm{m}}^{-2/3}$, $1\times {10}^{-15} \,{\mathrm{m}}^{-2/3}$, and $1\times {10}^{-14} \,{\mathrm{m}}^{-2/3}$, respectively. As shown in Fig. 7c, a model trained in a turbulence environment with a higher ${C}_{n}^{2}$ shows better adaptability. The models trained with a data set of ${C}_{n}^{2}=1\times {10}^{-14} \,{\mathrm{m}}^{-2/3}$ achieve almost 100% recognition accuracy except for one case (96.8%) of ${C}_{n}^{2}=1\times {10}^{-15} \,{\mathrm{m}}^{-2/3}$ and $\Delta l=0.05$. The results demonstrate that despite the strong AT level and the consequent collapse of the field structure, the ATANN can learn information on the unperturbed field pattern by discovering intrinsic local features that compose each fractional OAM mode from collected data. Additionally, we think that the relatively low accuracy at $\Delta l=0.05$, described above, can be improved with a hybrid training set considering various turbulence levels^23^.

It is necessary to discuss the appropriate mode spacing for the future applications of the fractional OAM beams in free-space communications. The minimum spacing is determined by the resolution of a deep-learning classifier, but it is limited and changed according to the strength of environmental factors such as AT level; see Fig. 6a. The laser beams propagating through atmospheric media experience different phase distortions depending on their wavelength and beam size^16^. So, it is proper to use the phasefront distortion $D/{r}_{0}$, where $D$ is the beam diameter, and ${r}_{0}={\left[0.423{k}_{0}^{2}{C}_{n}^{2}L\right]}^{-3/5}$ is the coherence length, for providing comprehensive criteria^16,30^. For turbulence environments that gives rise to the phasefront distortion of $<0.62$, the proposed scheme can exploit the mode spacing of 0.05. For the phase distortion ranging from 0.62 to 1.63, the mode spacing of 0.15 is available. In the case with $D/{r}_{0}$ between 1.63 and 2.47, one could select 0.20 as the mode interval. Here, we calculated the phasefront distortion using the Gaussian beam diameter, but actual beam sizes increase with the OAM order^14^. Therefore, the higher-order OAM beams experience stronger distortion than lower-order beams. Moreover, such problems become apparent and unavoidable as the number of OAM modes used increases. Therefore, for strong turbulence environments with $D/{r}_{0}>2.47$, one might apply uneven mode spacing for high-order OAM modes, e.g., a gap increasing with the OAM order. In addition, phase correction methods based on deep learning^26,27^ or a wavefront sensor^44^ could be considered, in conjunction with increasing the spacing.

### Stability enhancement using data augmentation

Data augmentation is a technique to artificially create new data from a given training set in the training phase of neural networks, which is implemented by applying various transformations, such as translation, rotation, and scaling^24,45^. Data augmentation randomly sets the degree of these transformations every epoch, effectively improving the generalization performance of neural networks. Here, we apply data augmentation with additive white Gaussian noise for modeling signal distortion due to sensor noise and investigate the change in the recognition accuracy over the signal-to-noise ratio (SNR). In order to make the model experience various noise levels, the algorithm generates a random number from a Gaussian distribution every epoch and applies it as the noise strength. The standard deviation of the distribution used in this work is 0.02, which corresponds to ~ 20 dB SNR.

**Figure 8:**
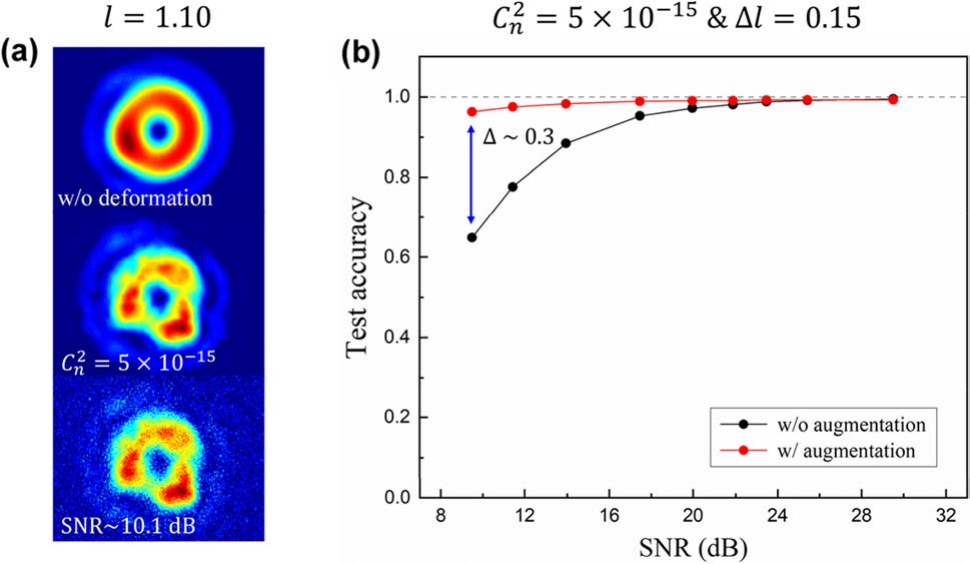
Data augmentation for enhancing noise tolerance. (**a**) Deformation of field distribution caused by AT (middle) and additive Gaussian noise (bottom). (**b**) Recognition accuracy against SNR. Black (red) circles show the result of a model trained without (with) data augmentation. Here, the SNR represents the average value for each test set.

As shown in Fig. 8a, we prepared noisy images (1,000 images per mode) to be used as test sets. Each noisy image is generated by adding random matrix of Gaussian distribution, which can be written as

8 $$\begin{array}{c}{I}_{\mathrm{noise}}\left(m,n\right)=I\left(m,n\right)+{\sigma }_{n}h\left(m,n\right),\end{array}$$

where $I\left(m,n\right)$ is a noiseless image, ${\sigma }_{n}$ is the strength of the added noise, and $h\left(m,n\right)$ is a Gaussian random matrix with mean 0 and standard deviation 1. SNR of each noisy image is calculated as^32^

9 $$\begin{array}{c}SNR=10{\mathrm{log}}_{10}\left(\frac{\overline{I}}{{\sigma  }_{n}}\right),\end{array}$$

where $\overline{I }$ represents the mean pixel value of the noiseless image $I\left(m,n\right)$. Figure 8b shows the measured test accuracy against SNR. Even if the same ${\sigma }_{n}$ is applied, there is some difference in the SNR of generated images. Thus, we used the average SNR per data set to display test results. Here, “w/ augmentation” represents the accuracy of a model trained by applying the data augmentation that adds Gaussian noise to input images. Whereas the recognition accuracy of the plain model starts to drop at 25 dB and rapidly decreases to 64.9%, the model using the data augmentation maintains the accuracy of at least 96% regardless of the applied noise strength. In particular, it maintains the accuracy of more than 99% up to 20 dB, which demonstrates that the proposed ATANN can be highly resistant to signal noise as well as AT through data augmentation.

### Application to multiplexed fractional OAM beams

**Figure 9:**
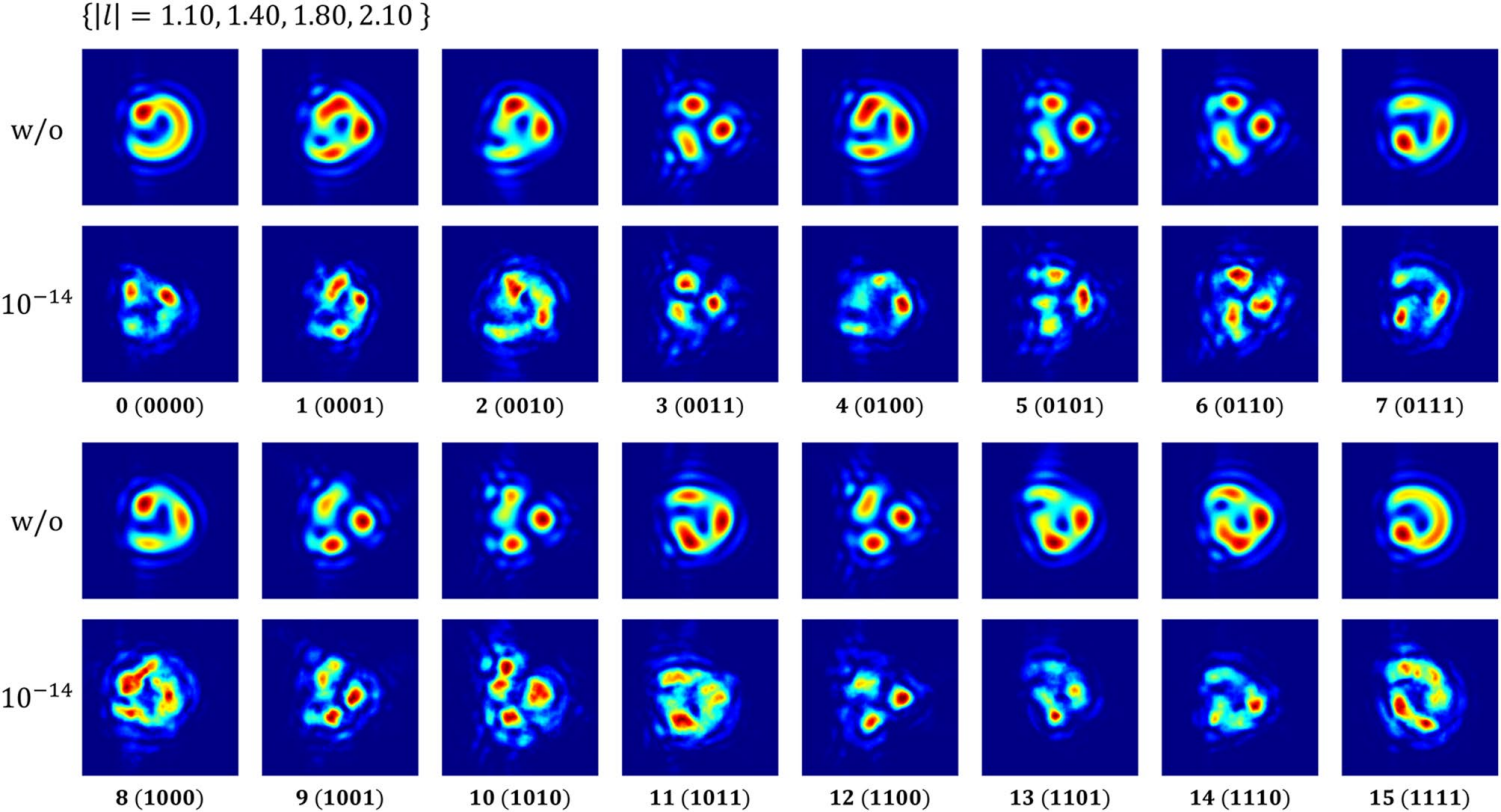
Intensity profiles of the received multiplexed fractional beams over 1000-m free-space channels with and without AT. Each label below a sub-image represents a 16-ary number and the bit string that corresponds to that number. In the presented scheme, 0 or 1 is assigned to each bit according to the sign of fractional OAMs constituting a superimposed mode.

A small interval of fractional modes makes more modes available despite the limited physical resources, but data encoding with a single OAM mode still has a limit on representing information of a large number of bits. For example, ${2}^{N}$ fractional OAM modes are required to encode *N*-bit data, and additional ${2}^{N}$ modes should be introduced for *N* + 1 bits. Therefore, the use of multiplexed fractional beams and their demodulation are explored. Here, the fractional mode set $\left\{\left|l\right|=1.10, 1.40, 1.80, 2.10\right\}$ is used to represent 4-bit data. 0 or 1 is assigned according to the sign of each mode, e.g., 0011 for $\left\{-1.10, -1.40, 1.80, 2.10\right\}$ and 0100 for $\left\{-1.10, 1.40, -1.80, -2.10\right\}$. The measured intensity profiles after 1000-m propagation in free-space channels with and without AT are shown in Fig. 9. The recognition accuracy for the multi modes is presented in Table 3, of which the first two columns show the results of single-mode schemes (10 integer modes from 1 to 10 and 10 fractional modes from 1.10 to 2.90). All tests presented here were conducted at the same turbulence level (${C}_{n}^{2}=1\times {10}^{-14} \,{\mathrm{m}}^{-2/3}$). The recognition accuracy of integer OAM beams was less than 90% despite their wide mode spacing, which is because the size feature for identification, such as radius, is weakened by AT^23^. On the other hand, the accuracy for both fractional schemes was measured to be over 99%. The results indicate that the multi-mode fractional OAM system can encode more bits with smaller $l$ values while maintaining recognition performance. Note that the broader the range of OAM, the more modes are available. Besides, one can increase further the amount of transmitted data by combining other photonic degrees of freedom.

**Table 3:** Measure recognition accuracy for different mode sets.

Mode set	Integer $\Delta l=1.00$	Fractional $\Delta l=0.20$	Multiplexed
Accuracy (%)	87.3	99.2	99.3

## Conclusion

In conclusion, we proposed and demonstrated deep-learning-based adaptive demodulation of fractional OAM modes distorted by AT. First, to prepare datasets for training and testing the designed neural network, we modeled 1-km turbulence channels with random phase screens emulating turbulence effects and simulated beam propagation through the channels. After that, we investigated the classification performance of the trained ATANN for 5 kinds of 10-ary fractional OAM systems (5 different mode intervals) and 5 kinds of AT levels (5 different ${C}_{n}^{2}$ values). Despite the strong turbulence level and the resulting collapse of the field structure, the ATANN achieved recognition accuracy of more than 99.2% for 10-ary fractional OAM systems with the mode spacing of $\ge 0.20$. For optical channels with weak and moderate turbulence strength levels, transmitted OAM modes were accurately identified regardless of mode intervals. Furthermore, we investigated the generalization ability of the ATANN for unknown turbulence levels. Our results showed that a model trained with a higher level of turbulence strength accommodates a wide range of turbulence environments, providing better adaptability. In addition to the turbulence effect, we applied the data augmentation technique for enhancing noise tolerance and demonstrated stable performance over a wide SNR range. The proposed scheme capable of performing high-resolution recognition despite external perturbations will offer reliable optical systems for free-space communications employing fractional OAM beams as data carriers. Moreover, the application of multiplexed fractional OAM beams might be helpful to realize higher data rates.
